# Intradiscal application of rhBMP-7 does not induce regeneration in a canine model of spontaneous intervertebral disc degeneration

**DOI:** 10.1186/s13075-015-0625-2

**Published:** 2015-05-27

**Authors:** Nicole Willems, Frances C Bach, Saskia G M Plomp, Mattie HP van Rijen, Jeannette Wolfswinkel, Guy CM Grinwis, Clemens Bos, Gustav J Strijkers, Wouter JA Dhert, Björn P Meij, Laura B Creemers, Marianna A Tryfonidou

**Affiliations:** Department of Clinical Sciences of Companion Animals, Faculty of Veterinary Medicine, Yalelaan 108, 3584 CM Utrecht, The Netherlands; Department of Orthopaedics, University Medical Center, Heidelberglaan 100, 3584 CX Utrecht, The Netherlands; Department of Pathobiology, Faculty of Veterinary Medicine, Yalelaan 1, 3584 CL Utrecht, The Netherlands; Department of Radiotherapy, University Medical Center, Heidelberglaan 100, 3584 CX Utrecht, The Netherlands; Department of Biomedical Engineering, University of Technology (TU/e), P.O. Box 513, 5600 MB Eindhoven, The Netherlands

## Abstract

**Introduction:**

Strategies for biological repair and regeneration of the intervertebral disc (IVD) by cell and tissue engineering are promising, but few have made it into a clinical setting. Recombinant human bone morphogenetic protein 7 (rhBMP-7) has been shown to stimulate matrix production by IVD cells *in vitro* and *in vivo* in animal models of induced IVD degeneration. The aim of this study was to determine the most effective dose of an intradiscal injection of rhBMP-7 in a spontaneous canine IVD degeneration model for translation into clinical application for patients with low back pain.

**Methods:**

Canine nucleus pulposus cells (NPCs) were cultured with rhBMP-7 to assess the anabolic effect of rhBMP-7 *in vitro*, and samples were evaluated for glycosaminoglycan (GAG) and DNA content, histology, and matrix-related gene expression. Three different dosages of rhBMP-7 (2.5 μg, 25 μg, and 250 μg) were injected *in vivo* into early degenerated IVDs of canines, which were followed up for six months by magnetic resonance imaging (T2-weighted images, T1rho and T2 maps). Post-mortem, the effects of rhBMP-7 were determined by radiography, computed tomography, and macroscopy, and by histological, biochemical (GAG, DNA, and collagen), and biomolecular analyses of IVD tissue.

**Results:**

*In vitro*, rhBMP-7 stimulated matrix production of canine NPCs as GAG deposition was enhanced, DNA content was maintained, and gene expression levels of *ACAN* and *COL2A1* were significantly upregulated. Despite the wide dose range of rhBMP-7 (2.5 to 250 μg) administered *in vivo*, no regenerative effects were observed at the IVD level. Instead, extensive extradiscal bone formation was noticed after intradiscal injection of 25 μg and 250 μg of rhBMP-7.

**Conclusions:**

An intradiscal bolus injection of 2.5 μg, 25 μg, and 250 μg rhBMP-7 showed no regenerative effects in a spontaneous canine IVD degeneration model. In contrast, intradiscal injection of 250 μg rhBMP-7, and to a lesser extent 25 μg rhBMP-7, resulted in extensive extradiscal bone formation, indicating that a bolus injection of rhBMP-7 alone cannot be used for treatment of IVD degeneration in human or canine patients.

**Electronic supplementary material:**

The online version of this article (doi:10.1186/s13075-015-0625-2) contains supplementary material, which is available to authorized users.

## Introduction

Low back pain is one of the major causes of disability in humans [1]. Several studies have provided evidence for its association with intervertebral disc (IVD) degeneration [2,3]. Current therapies, such as physiotherapy, anti-inflammatory medications, and surgery alleviate symptoms, but do not restore the physiological function of the degenerated IVD. Prevention of further degeneration or regeneration of the IVD requires intervention at an early stage. Strategies for biological repair and regeneration of the IVD by cell and tissue engineering are promising, but are not widely clinically applicable thus far. A number of studies have been performed on bone morphogenetic proteins (BMPs), given their potential regenerative role in degenerative IVD disease [4]. BMPs belong to the transforming growth factor-β (TGF-β) superfamily and are involved in many developmental processes [4,5]. Recombinant human bone morphogenetic protein 7 (rhBMP-7) has been tested extensively and appears to be a promising BMP for IVD regeneration [6-8], as it has been shown to have beneficial effects on extracellular matrix production of rabbit, bovine, and human IVD cells *in vitro* [7,9-13]. Several animal models with experimental IVD degeneration were used to study the efficacy and translational aspects of BMP-7 towards a clinical application in humans [14]. In rabbits with induced IVD degeneration, rhBMP-7 restored disc height and improved the IVD viscoelastic properties by increasing the proteoglycan content [15,16]. An anti-catabolic effect of rhBMP-7 was shown in a rat model with induced IVD degeneration [17]. Also in a canine model of allogenic IVD transplantation, nucleus pulposus cells (NPCs) expressing rhBMP-7 prevented degeneration of the transplanted IVD at six months follow-up [18]. Thus far, novel regenerative therapies involving intradiscal administration of rhBMP-7 have been tested in animal models with induced IVD degeneration, but not in an animal model with spontaneous IVD degeneration that more closely resembles the biological condition in humans. Furthermore, dose response studies evaluating intradiscal injection of rhBMP-7 and its possible adverse effects are not available [4,19].

The goal of this study was to assess the effect of a wide range of doses of intradiscal injections of rhBMP-7 (2.5 to 250 μg) in a canine model with spontaneous IVD degeneration that closely resembles human IVD degeneration and disease [20,21]. For this, we first investigated the anabolic effect of two dosages of rhBMP-7 on early degenerated canine NPCs *in vitro*. Potential regenerative effects of rhBMP-7 *in vivo* were studied by obtaining conventional T2-weighted images and T2 and T1ρ maps in a longitudinal manner. Both T1ρ and T2 relaxation times are correlated with IVD degeneration since T2 relaxation times correlate strongly with water content, while T1ρ relaxation times are particularly sensitive to a decrease in glycosaminoglycan (GAG) content in the NP [22,23]. At six months follow-up, the effects of rhBMP-7 were determined post-mortem by radiography, computed tomography, and macroscopy, and by histological, biochemical, and biomolecular analyses.

## Methods

### Ethics statement

All procedures involving animals were approved and conducted in accordance with the guidelines set by the Animal Experiments Committee (DEC) of Utrecht University (experimental numbers: 2012.III.07.065, 2013.III.02.017, and 2013.II.12.126), as required by Dutch regulation.

### Isolation and culture of nucleus pulposus cells

NP tissue was separated from early degenerated IVDs (Pfirrmann grade 2) of 12 laboratory beagle dogs; care was taken to avoid the transitional zone. Tissue was washed with high-glucose (hg)-DMEM + GlutaMAX™ + pyruvate (31966, Invitrogen, Paisley, UK) + 1% penicillin/streptomycin (p/s) (P11-010, PAA Laboratories GmbH, Piscataway, NJ, USA), and digested with 0.15% pronase (11459643001, Roche Diagnostics, IN, USA) for 45 minutes at 37°C, and subsequently digested overnight with 0.15% collagenase II (4176, Worthington, Lakewood, NJ, USA) at 37°C. NPCs were filtered over a 70-μm filter, centrifuged (5 minutes at 500 g), and cryopreserved at passage 0 (hg-DMEM + GlutaMAX + 10% dimethyl sulfoxide (DMSO) + 10% fetal bovine serum (FBS) (high performance 16000–044, Gibco, Bleiswijk, The Netherlands)) until further use. NPCs were expanded in expansion medium (hg DMEM + GlutaMAX + pyruvate (Invitrogen), 10% FBS, 1% p/s, 20 mM ascorbate-2-phosphate (A8960, Sigma-Aldrich, Saint Louis, MO, USA), 0.02 mM dexamethasone (D1756, Sigma-Aldrich), 1 ng/ml basic fibroblast growth factor (bFGF) (PHP105, AbD Serotec, Oxford, UK), and 0.5% Fungizone (15290–018, Invitrogen) in 175-cm^2^ cell culture flasks (660175, Greiner bio-one, Cellstar, Alphen aan den Rijn, The Netherlands) until passage 2 (P2).

Cells (P2) were pooled to yield 5 different NPC donor groups and pelleted in ultra-low attachment 96-well plates (Corning Costar 7007, Sigma-Aldrich) by centrifugation at 185 g for 8 minutes. Each pellet contained 200,000 NPCs and was cultured in 200 μl chondrogenic culture medium for 28 days (5% CO_2_, 20% O_2_). Standard chondrogenic medium (hg-DMEM + GlutaMAX (Invitrogen), was supplemented with 1% insulin-transferrin-selenium (ITS)  + premix (354352 Corning, Tewksbury, MA, USA), 20 mg/ml proline (P5607 Sigma-Aldrich), 1% p/s, 0.5% Fungizone, 0.02 mM dexamethasone, and 20 mM ascorbate-2-phosphate), and remained untreated (negative control) or was supplemented with 10 or 100 ng/ml rhBMP-7 (mammalian cell derived; 354-BP-010 R&D Systems Europe Ltd, Oxon, UK). Media were renewed twice weekly, collected per week, and stored at −80°C for analysis of GAG content.

### Glycosaminoglycan and DNA content of nucleus pulposus cell pellets and glycosaminoglycan content of culture media

At days 7 and 28, two NPC pellets per donor and condition were digested overnight at 60°C in papain (250 μg/ml papain (P3125, 100 mg, Sigma-Aldrich) + 1.57 mg cysteine HCL (C7880, Sigma-Aldrich)). The 1,9-dimethylmethylene blue (DMMB) assay was used to quantify GAG content [24] of the pellets and media. GAG concentrations were calculated by using chondroitin sulfate from shark cartilage (C4384, Sigma-Aldrich) as a standard and the absorbance was read at 540/595 nm. The Quant-iT™ dsDNA Broad-Range assay kit in combination with a Qubit™ fluorometer (Invitrogen) was used in accordance with the manufacturer’s instructions to determine the DNA content of the NPC pellets.

### RNA isolation and quantitative RT-PCR of nucleus pulposus cell pellets

At days 7 and 28, RNA was isolated from two pellets per donor and condition and pooled. After crushing the pellets with a pellet pestle (9951–901, Argos Technologies, Elgin, IL, USA), total RNA was isolated by using the RNeasy Micro Kit according to the manufacturer’s instructions. After on-column DNase-I digestion (RNase-free DNase kit; Qiagen, Venlo, The Netherlands), RNA was quantified by using a NanoDrop 1000 spectrophotometer (Isogen Life Science, IJsselstein, The Netherlands). The iScript™ cDNA Synthesis Kit (Bio-Rad, Veenendaal, The Netherlands) was used to synthesize cDNA.

Quantitative PCR (qPCR) was performed using an iCycler CFX384 Touch thermal cycler, and IQ SYBRGreen Super mix (Bio-Rad). qPCR was performed to assess the effects of rhBMP-7 at gene expression levels on: 1) extracellular matrix anabolism (aggrecan (*ACAN*), collagen type 2 alpha 1 (*COL2A1*), collagen type 1 alpha 1 (*COL1A1*)), 2) proliferation (cyclin-D1 (*CCND1*)), 3) extracellular matrix catabolism (a disintegrin and metalloproteinase with thrombospondin motifs (*ADAMTS5*), matrix metalloproteinase 13 (*MMP13*), tissue inhibitor of metalloproteinase 1 (*TIMP1*)), 4) apoptotic markers (B-cell lymphoma 2-associated X (*BAX*), B-cell lymphoma 2 (*BCL2*), caspase 3 (*CASP3*)), and 5) BMP signaling (BMP receptor 1A (*BMPR1A*), BMP receptor 1B (*BMPR1B*), BMP receptor 2 (*BMPR2*), inhibitor of DNA binding 1 (*ID1*), noggin (*NOG*))*.*

Details on the primer pairs employed are given in Additional file 1. All dog-specific primers were designed in-house using Perlprimer [25], except for *MMP13* [26]. Primer specificity was evaluated with BLAST, and the designed amplicon was tested for secondary structures using MFold [27]. Primers were purchased from Eurogentec (Maastricht, The Netherlands). Amplification efficiencies ranged from 86 to 119%. Relative expression levels were determined by the efficiency-corrected delta-delta CT (ΔΔCt) method. Ct values of each target gene were normalized by the mean Ct value of four reference genes (ΔCt), that is, glyceraldehyde 3-phosphate dehydrogenase (*GAPDH*), ribosomal protein S19 (*RPS19*), *s*uccinate dehydrogenase complex, subunit A, flavoprotein variant (*SDHA*), and hypoxanthine-guanine phosphoribosyltransferase (*HPRT*), whereas the mean Ct of all conditions for each target gene was used as a calibrator (ΔΔCt).

### Histopathological evaluation of nucleus pulposus cell pellets

Safranin-O/fast green staining was performed to evaluate the presence of GAG deposition at a histopathological level. Two pellets per donor and condition were fixed overnight in neutral buffered formaldehyde 4% (Boom BV, Meppel, The Netherlands) supplemented with 1% eosin (115935, Merck, Schiphol-Rijk, The Netherlands). Subsequently, the pellets were embedded in alginate and then paraffin. Sections (5 μm) were stained with Mayer’s hematoxylin (3870, Avantor Performance Materials, Center Valley, PA, USA), safranin-O (58884, Sigma-Aldrich) and, as a counterstaining, fast green (F7252, Sigma-Aldrich).

### Experimental animals

Seven intact male beagle dogs with a median age of 1.3 years (range: 1.1 to 1.8) and a median weight of 11.7 kg (range: 10.2 to 12.8) were purchased from Harlan (Gannat, France). All dogs underwent a general, orthopedic, and neurologic examination by a board-certified veterinary surgeon (BM). The study was set up following a randomized block design. Single bolus injections of 2.5 μg, 25 μg, and 250 μg rhBMP-7 (mammalian cell derived, CYT-276, ProSpec-Tany TechnoGene Ltd, Ness-Ziona, Israel), and a sucrose buffer (sham) were injected into the NPs in the T13-S1 spinal segments in a balanced Latin square design. IVDs adjacent to those injected with 250 μg of rhBMP-7 remained untreated. Preliminary studies in cadaveric spines showed that a volume of 40 μl could be injected into the NP without considerable resistance (Meij BP, Tryfonidou MA, Willems N). The highest dosage of rhBMP-7 was constrained by the highest possible concentration that could be accomplished via dialysis, that is, 250 μg in 40 μl.

### Preparation of rhBMP-7 for *in vivo* application

Prior to the *in vivo* studies, the biological activity of rhBMP-7 from different manufacturers was determined in an alkaline phosphatase (ALP) activity assay in ATDC5 cells, amongst them rhBMP-7 from R&D and ProSpec-Tany. rhBMP-7 from ProSpec-Tany showed the highest biological activity, as shown in Additional file 2, and was further chosen to be employed in the *in vivo* studies.

RhBMP-7 (CYT-276, ProSpec-Tany TechnoGene Ltd) was reconstituted in 60 μl sterile water. This solution was dialyzed against a buffer solution containing 17% sucrose, 20% mannitol, 332 mM glycine, and 0.8% Tween 20, using a slide-A-Lyzer dialysis cassette (66454, Thermo Fisher Scientific Inc., Rockford, IL, USA) with a molecular weight cutoff of 10 kDa overnight with 4 buffer changes. The final solution containing 300 μg of rhBMP-7 (calculated amount) was freeze-dried overnight and reconstituted in sterile water, to achieve a final concentration of 250 μg rhBMP-7 in 40 μl buffer solution containing 1% sucrose, 1.2% mannitol, 20 mM glycine, and 0.05% Tween 20. Activity of the dialysate was shown to be retained *in vitro* through its capacity to induce ALP production in mice ATDC5 cells. Additional file 2 shows the biologic activity of the dialyzed rhBMP-7 compared with the pre-dialyzed rhBMP-7.

### Magnetic resonance imaging

Magnetic resonance imaging (MRI) of the lumbar vertebral column were performed in fully anesthetized dogs prior to surgery (t_0_) and at 6 (t_6_), 12 (t_12_), and 24 (t_24_) weeks after surgery. Dogs were pre-medicated with intravenous dexmedetomidine 10 μg/kg intravenously and butorphanol 0.1 mg/kg, and anesthesia was induced with a continuous rate infusion of propofol (3 to 4 mg/kg) and dexmedetomidine 1 μg/kg. A laryngeal mask was inserted, and anesthesia was maintained with isoflurane (2 to 3%) in a 1:1 oxygen/air mixture. Prior to the first MRI scan, a blood sample was drawn from the jugular vein to assess white blood cell count and differentiation, to exclude systemic inflammation. MRI was performed with a 1.5 Tesla scanner using a spine array coil (Philips Healthcare, Best, The Netherlands). Sagittal T2-weighted (T2W) images were acquired using a turbo-spin echo (TSE) pulse sequence with the following parameters: repetition time (TR) = 2,557 ms, echo time (TE) = 100 ms, field of view (FOV) = 200 mm, acquisition matrix = 332 × 306, slice thickness = 2 mm, number of slices = 13. For T2 mapping, acquisition parameters were as follows: TR = range 2,000 to 4,000 ms, TE = 12.5 to 100 ms in 12.5 ms increments, FOV = 250 mm, acquisition matrix = 416 × 200, slice thickness = 2 mm, number of slices = 11. Sagittal T1ρ weighted imaging was performed using a spin-lock-prepared sequence with a three dimensional multi-shot ultrafast gradient echo (T1-TFE) readout with the following parameters: TR = 8.3 ms, TE = 4.3 ms, FOV = 250 mm, acquisition matrix = 416 × 378, slice thickness = 2 mm, number of slices = 25, TFE factor = 64, flip angle = 10°, shot interval = 2,000 ms. To allow quantitative T1ρ mapping, data were acquired five times, each with a different spin-lock time (TSL): 1, 10, 20, 40, and 80 ms. Spin-lock amplitude was set to 500 Hz.

### T2 and T1ρ quantification

Mid-sagittal slices of T2W images were used to evaluate the grade of degeneration at all time points. Lumbar IVDs were assessed by a veterinary radiologist that was blinded to treatment allocation, according to the Pfirrmann classification validated for dogs by Bergknut *et al*. [28]. Only lumbar IVDs with a Pfirrmann score II at t_0_ were included for injection. Disc height index (DHI) was calculated at all time points on T2W images for each IVD, according to the method described by Masuda *et al*. [15]. In short, DHI was calculated by averaging widths of the dorsal, middle, and ventral parts of the vertebral disc and dividing that by the average of dorsal, middle, and ventral body heights of the adjacent cranial and caudal vertebrae. To calculate the DHI of L7-S1, the average body height of only the cranial vertebra (L7) was used, as S1 has a different shape than the lumbar vertebrae. For the analysis of T2 and T1ρ values, an oval-shaped region of interest (ROI) was manually segmented on mid-sagittal sections, to select NP tissue in each IVD in the free open-source DICOM viewer Osirix (Pixmeo, Geneva, Switzerland). ROIs were exported to and analyzed with Wolfram Mathematica 10.0 (Wolfram Research, Champaign, IL, USA). T2 and T1ρ values were computed by calculating the mean signal intensity (S) in each ROI, and by fitting these intensity data into the following equations, respectively, using the Levenberg-Marquardt nonlinear least-squares method implemented in Mathematica:

1 $$ \mathrm{S}\left(\mathrm{T}\mathrm{E}\right) = {\mathrm{S}}_0\mathrm{e}{\hbox{-}}^{\mathrm{TSL}/\mathrm{T}2} $$

or

2 $$ \mathrm{S}\left(\mathrm{T}\mathrm{S}\mathrm{L}\right) = {\mathrm{S}}_0\mathrm{e}{\hbox{-}}^{\mathrm{TE}/\mathrm{T}1\uprho} $$

S_0_ denotes the equilibrium magnetization, whereas S(TSL) and S(TE) indicate the T2- and T1ρ-prepared signals, respectively*.*

### Surgical procedure

The anesthesia protocol for MRI and surgery was identical. Analgesia was provided pre-operatively by intravenous methadone 0.5 mg/kg and carprofen 4 mg/kg, and perioperatively by a continuous rate infusion of a combination of fentanyl (loading dose 10 μg/kg, 15 to 20 μg/kg/hr) and ketamine (loading dose 0.5 mg/kg, 10 μg/kg/min). Dogs were positioned in right recumbence to expose and inject T13-L6 via a left lateral approach, and subsequently in ventral recumbence to expose and inject L6-S1 via a dorsal approach. A 100 μl gastight syringe (7656–01 Model 1710 RN) with a 27 G needle (25 mm, 12° beveled point; Hamilton Company USA, Reno, Nevada, USA) was used to inject 40 μl of the BMP-7 containing solutions or control into the NP under magnified vision (3.3×). Location of the tip of the needle in the NP was estimated by the distance of passage through the annulus fibrosus (AF) (1 cm), while encountering steady resistance. When the NP was reached, the resistance decreased and the volume of 40 μl could be easily injected. The needle was retracted slowly to allow the AF puncture site to close, and the site was inspected for extrusion of the administered compound and rinsed with 0.9% NaCl. Postoperative pain management in all dogs consisted of intramuscular methadone 0.3 mg/kg every 6 hours and subcutaneous carprofen 4 mg/kg once a day during the first 24 hours, and tramadol 2 to 5 mg/kg administered orally four times a day, and carprofen 4 mg/kg administered orally for the following 7 and 10 days, respectively. All dogs were treated postoperatively with antibiotics (amoxicillin/clavulanic acid 12.5 mg/kg twice a day) for five days. Dogs were monitored daily throughout the study to assess pain symptoms according to the short form of the Glasgow composite pain scale [29]. Dogs that showed signs of pain received carprofen and/or tramadol.

### Radiographic imaging and computed tomography

Radiographs and computed tomography (CT) scans of the T11-S3 segment were obtained postmortem (t_24_) and were evaluated by an independent veterinary radiologist for new bone formation, end plate sclerosis, and disc protrusion (only CT). Lateral and dorsoventral radiographs were obtained with a digital radiography system (Philips digital Rad TH, Eindhoven, The Netherlands) using 50 kVp and 5 mA. Transverse CT images were acquired with a third-generation single-slice helical CT-scanner (Philips Secura, Eindhoven, The Netherlands). Contiguous 2-mm thick slices with 1 mm overlap were obtained from T11-S3 with exposure settings of 120 kV and 260 mA.

### Sample collection, macroscopic grading, and histopathological grading

Dogs were euthanized 24 weeks post-injection by way of intravenous sedation with dexmedetomidine 0.04 mg/kg, followed by pentobarbital 200 mg/kg. Subsequently, the vertebral column (T12-S1) was harvested using an electric multipurpose saw (Bosch, Stuttgart, Germany). All muscles were removed and the lumbar vertebrae were transected transversely with a band saw (EXAKT tape saw, EXAKT Advanced Technologies GmbH, Norderstedt, Germany), resulting in nine spinal units (½ vertebra - endplate - IVD - endplate - ½ vertebra). A diamond band pathology saw (EXAKT 312 saw; EXAKT diamond cutting band 0.1 mm D64; EXAKT Advanced Technologies GmbH, Norderstedt, Germany) was used to transect these units sagittally into two identical parts. From one part, the endplate and vertebra were removed with a surgical knife, and the remaining IVD tissue containing NP and AF, was snap frozen in liquid nitrogen and stored at −80°C for biochemical and biomolecular analyses. The other part was photographed (Olympus VR-340, Hamburg, Germany) for macroscopic evaluation (Thompson score) and fixed in 50 ml of 4% buffered formaldehyde (Klinipath, Duiven, The Netherlands) at 4°C for 14 days. Images of the IVD segments were evaluated blind and in random order by two independent blinded investigators, according to the Thompson grading scheme, which has been validated in dogs [30].

Samples were decalcified in 35% formic acid and 6.8% sodium formate in a microwave oven (Milestone Microwave Laboratory Systems, Sorisole, Italy) overnight at 37°C, for 7 nights and embedded in paraffin [31]. Sections (5-μm thick) were stained with hematoxylin (109249, Merck) and eosin (115935, Merck) and with picrosirius red (saturated aqueous picric acid: 36011, Sigma-Aldrich; sirius red: 80115, Klinipath)/alcian blue (alcian blue: 05500, Sigma-Aldrich; glacial acetic acid: 100063, Merck) and evaluated according to the grading scheme of Bergknut *et al*. [21]. Histological sections were scored blind and in random order by two independent investigators (NW and SP) using an Olympus BX41 microscope. In case of disagreement, samples were also scored by a board-certified veterinary pathologist (GG).

### RNA isolation and qPCR of nucleus pulposus and annulus fibrosus

Cryosections (60-μm thick) of the IVD of the remaining spinal unit (endplate - IVD) were cut with a cryostat (Leica CM1800 cryostat, Leica Microsystems Inc., Bannockburn, USA) and collected on RNA-se free glass slides. The NP and AF tissues were separated and half of the tissues were collected in 400 μl and 750 μl cOmplete lysis M EDTA-free buffer (Roche Diagnostics Nederland BV, Almere, The Netherlands), respectively, and stored at −80°C until biochemical analysis. The other halves were collected in 300 μl Buffer RLT containing 1% β-mercapto-ethanol (Qiagen, Venlo, The Netherlands) and stored at −80°C until biomolecular analysis. Total RNA was isolated by using the RNeasy Fibrous Tissue Mini Kit (Qiagen, Venlo, The Netherlands). The incubation period with proteinase K was reduced to five minutes to increase RNA yield. RNA quantification and cDNA synthesis were performed in a similar way as described for the NPC pellets *in vitro*. Reference genes and the subset of target genes were similar to those determined *in vitro*.

### Glycosaminoglycan, DNA, and collagen assays of nucleus pulposus and annulus fibrosus

To measure GAG and DNA content, the NP and AF samples were homogenized in cOmplete lysis M EDTA-free buffer in a TissueLyser II (Qiagen) for 2× 30 seconds at 20 Hz. The supernatant and pellet of each NP and AF were digested overnight in papain and measurements were performed as described *in vitro*. Collagen was quantified in the pellets of the NP and AF by using a hydroxyproline assay [32]. Samples were freeze-dried overnight, hydrolyzed at 108°C overnight in 4 M NaOH, centrifuged (15 seconds at 14,000 g) and stored at −20°C. Prior to measurements, samples were centrifuged (15 seconds at 14,000 g) once more, chloramine T reagent (2426, Merck) was added, and samples were allowed to shake for 20 minutes at 170 rpm. Freshly prepared dimethylaminobenzaldehyde (3058 Merck) was added, and samples were incubated for 20 minutes at 60°C. The absorbance was read at 570 nm and collagen content was calculated from the hydroxyproline content by multiplying with a factor 7.5 [32]. DNA and collagen content in the supernatants were negligible and therefore not included in the calculations. Total GAG and collagen content were normalized to DNA content in the sample.

### Statistical analyses

All data were analyzed by using R statistical software, package 3.0.2 [33]. Residual plots and quantile-quantile (Q-Q) plots were used to check normality of the data. In case of violation, data were logarithmically transformed. If non-normality remained after transformation, nonparametric tests were employed.

*In vitro*, cumulative GAG release, GAG and DNA content, the GAG/DNA ratio, and ∆CT values for the investigated target genes, were statistically evaluated by using the nonparametric Kruskal-Wallis test, followed by the Mann–Whitney U-test. The effect of the injected treatments *in vivo* on GAG, DNA and collagen content, DHI, and T1ρ and T2 values was analyzed with a linear mixed-effect model. A random effect ‘dog’ (dog one to seven) was incorporated to capture the correlation between multiple measurements within one dog. For GAG, DNA, and collagen analysis, factors incorporated into the model as a fixed effect were ‘treatment’ (2.5 μg rhBMP-7, 25 μg rhBMP-7, 250 μg rhBMP-7, and sham), ‘tissue’ (NP and AF), and their interaction. For DHI analysis ‘treatment’, ‘time’ (t_0_, t_6_, t_12_, and t_24_), and their interaction served as fixed effect factors. The Cox proportional hazards regression model was used to estimate the effect of the injected treatments on gene expression levels *in vivo*. Calculations were performed on the ratio of the CT values for each target gene to the mean CT value of the reference genes. CT values of 40 or more were right censored. Regression coefficients were estimated by the maximum likelihood method. Model selection was based on the lowest Akaike Information Criterion (AIC). Differences between treatments were considered significant if the confidence interval did not include 0, whereas hazard ratios were considered significant if the confidence interval did not include 1. For *in vitro and in vivo* analyses, confidence intervals were calculated and stated at the 99% confidence level to correct for multiple comparisons. Significant differences and the corresponding confidence intervals are represented in Additional file 3.

## Results

### Effect of rhBMP-7 on early degenerated canine nucleus pulposus cell pellets *in vitro*

#### Cell maintenance and increased glycosaminoglycan release and content by rhBMP-7

**Figure 1:**
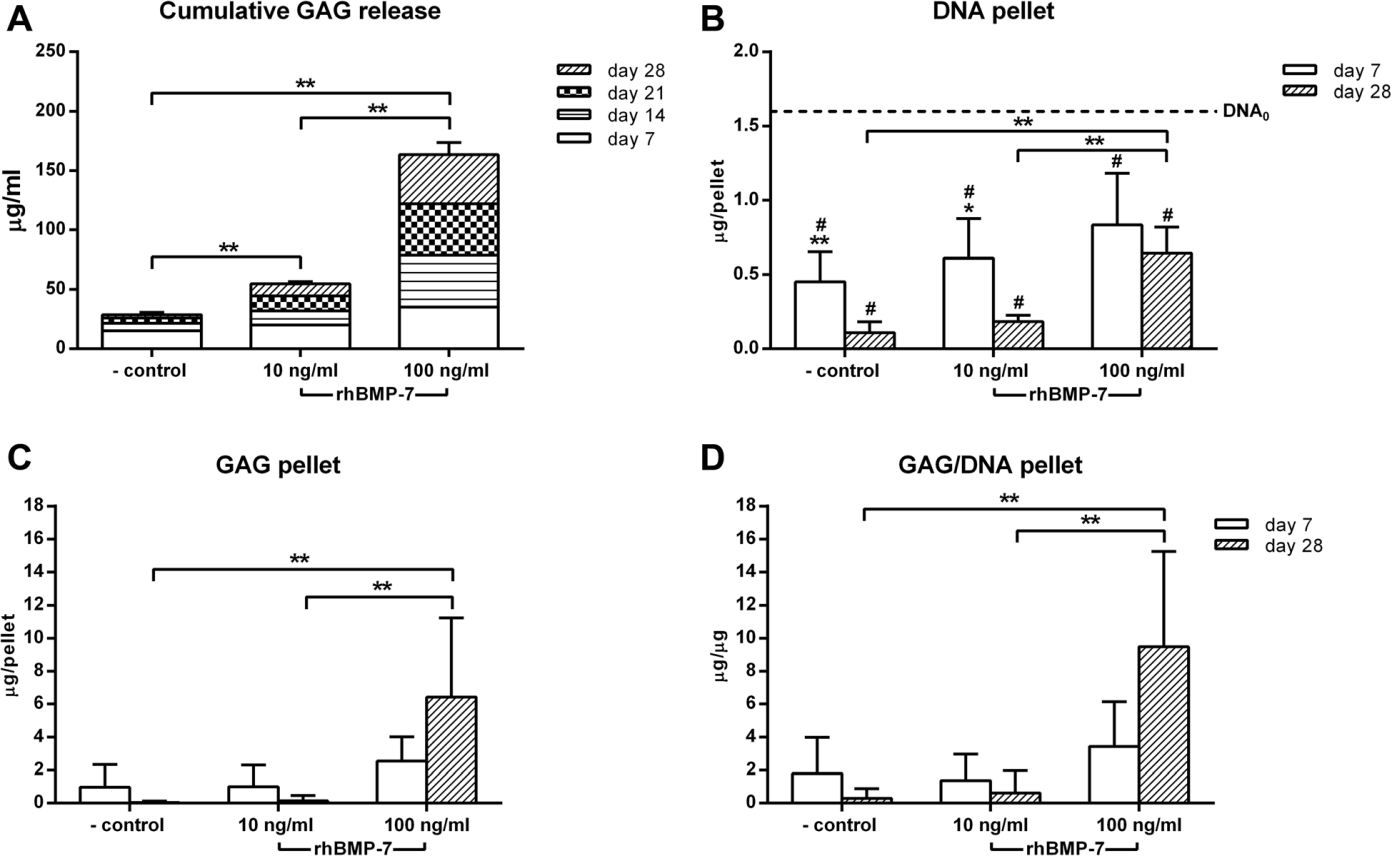
Glycosaminoglycan (GAG) release and GAG, DNA, and GAG/DNA content in cultured nucleus pulposus cells (NPCs) treated with 10 or 100 ng/ml rhBMP-7. **A**. NPC pellets treated with rhBMP-7 show a significant dose-dependent increase in cumulative GAG release into the medium compared with the negative control. **B**. Regardless of the culture condition, DNA content of the NPC pellets was significantly lower compared with DNA content at day 0 (DNA_0_; dashed line), indicated by #. NPC pellets treated with 100 ng/ml rhBMP-7 showed a significantly higher DNA content at day 28 compared with the negative control and the 10 ng/ml rhBMP-7-treated NPC pellets. **C**, **D**. A significant increase in GAG content and GAG/DNA at day 28 was shown in the 100 ng/ml rhBMP-7-treated NPC pellets compared with the negative control and the 10 ng/ml rhBMP-7-treated NPC pellets. Data are expressed as mean ± SD. **Indicates significant difference at a 99% confidence interval (CI); *indicates significant difference at a 98% CI; # indicates a significant difference at a 99% CI.

**Figure 2:**
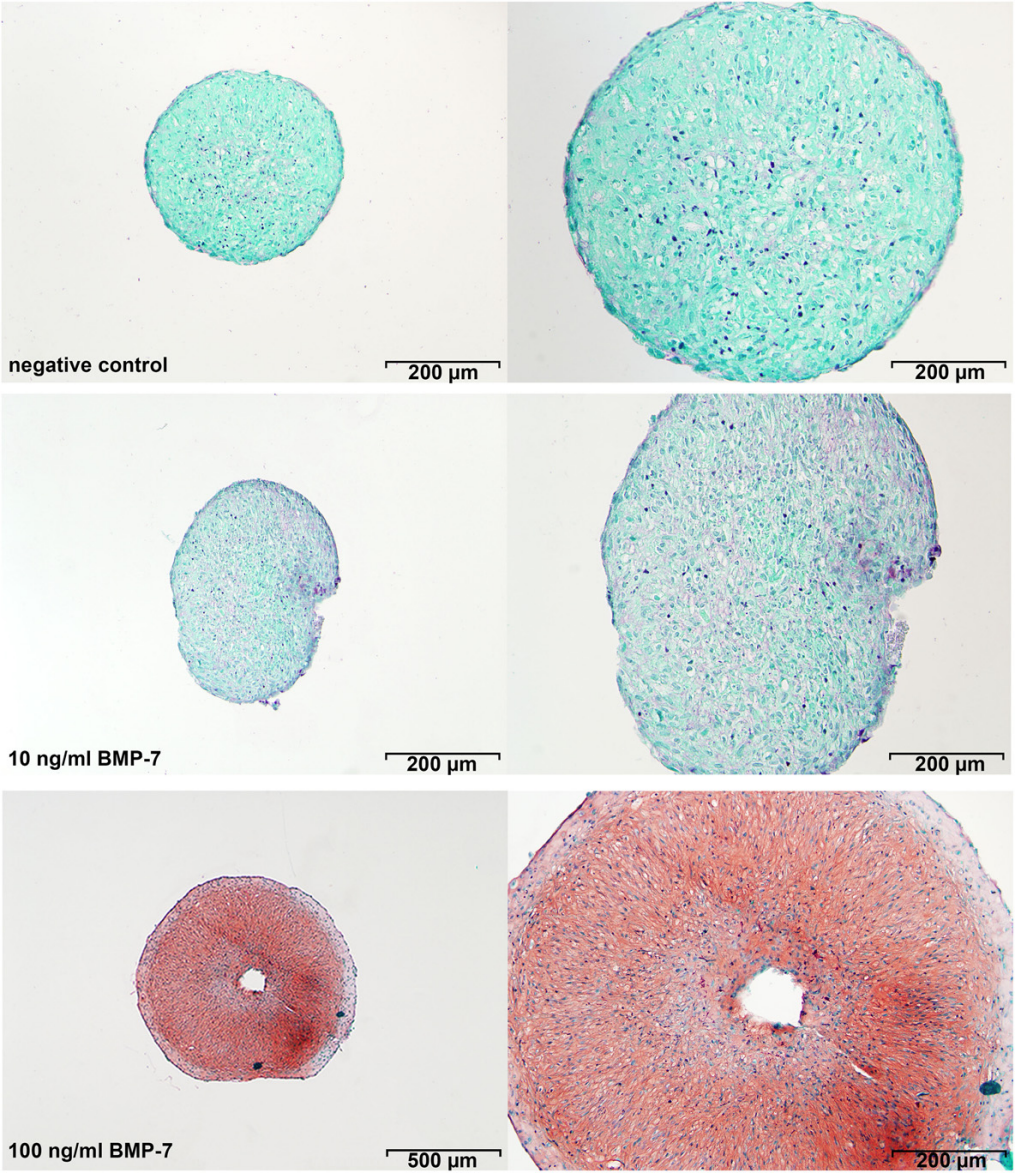
Representative histological images of nucleus pulposus cells (NPCs) cultured in pellets for 28 days stained with Safranin-O/fast green. NPC pellets treated with 100 ng/ml rhBMP-7 showed a positive Safranin-O/fast green staining for glycosaminoglycan (GAG) and an increased size compared with the negative control and the 10 ng/ml rhBMP-7-treated pellets.

NPC pellets treated with 10 and 100 ng/ml rhBMP-7 showed a dose-dependent significant increase in cumulative GAG release into the medium compared with the negative control (Figure 1A). Regardless of the culture condition, DNA content of the NPC pellets was significantly lower compared with DNA content at day 0. Treatment with 100 ng/ml rhBMP-7 resulted in a significantly higher DNA content at day 28 compared with the negative control and the 10 ng/ml rhBMP-7-treated NPC pellets (Figure 1B). A significant increase in GAG content and GAG/DNA at day 28 was shown in the 100 ng/ml rhBMP-7-treated NPC pellets compared with the negative control and the 10 ng/ml rhBMP-7-treated NPC pellets. Safranin-O/fast green staining of the NPC pellets at day 28 showed a higher GAG deposition in the matrix of the pellets treated with 100 ng/ml rhBMP-7 compared with the negative control and the 10 ng/ml rhBMP-7-treated pellets (Figure 2).

#### Pro-anabolic and anti-apoptotic effect of rhBMP-7

**Figure 3:**
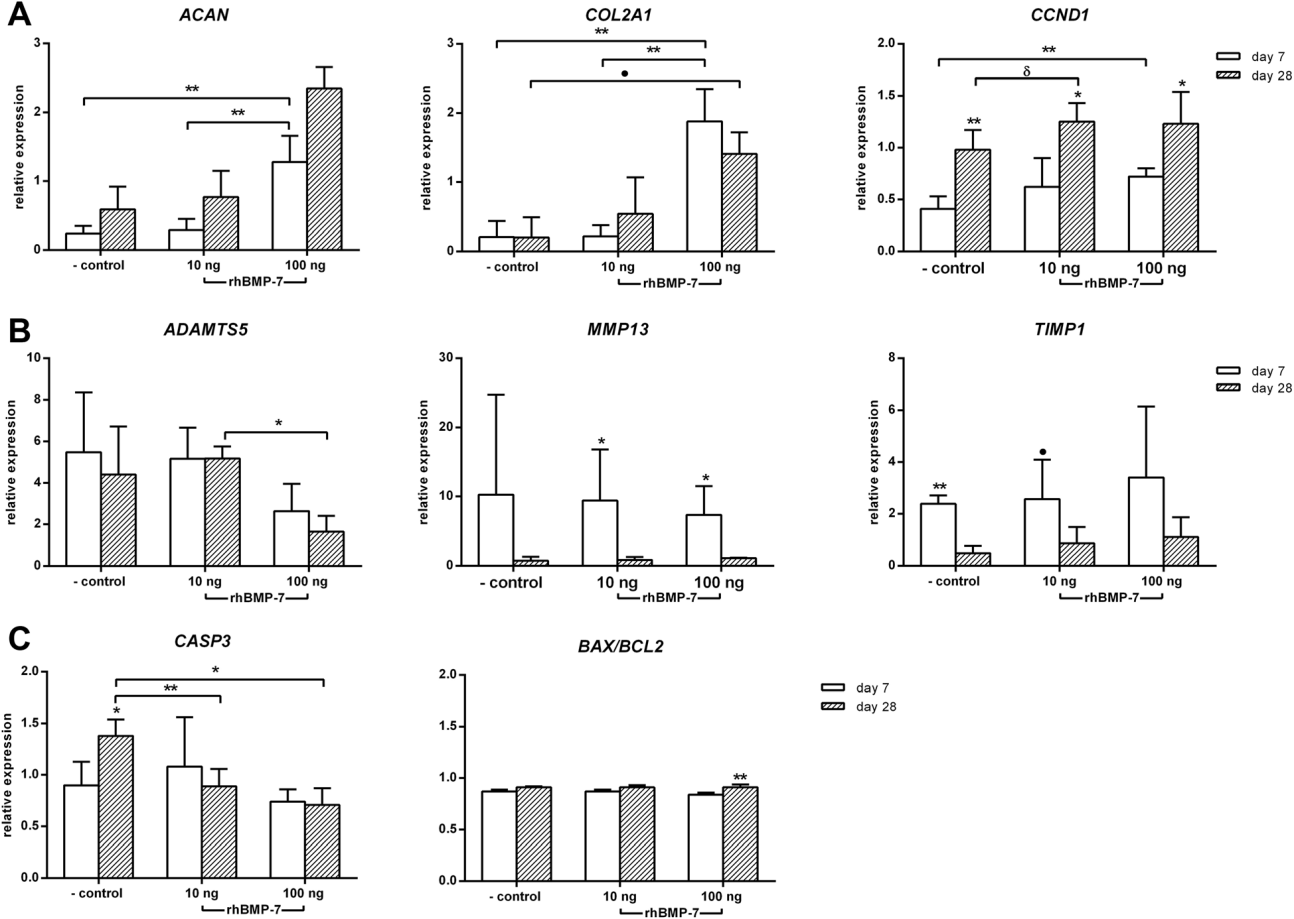
Relative gene expression of relevant target genes in nucleus pulposus cells (NPCs) cultured in pellets at day 7 and 28. **A**. Anabolic markers: aggrecan (*ACAN*), collagen type 2 alpha 1 (*COL2A1*), cyclin-D1 (*CCND1*); **B**. Catabolic markers: a disintegrin and metalloproteinase with thrombospondin motifs 5 (*ADAMTS5*), matrix metalloproteinase 13 (*MMP13*), tissue inhibitor of metalloproteinase 1 (*TIMP1*); **C**. Apoptotic markers: caspase 3 (*CASP3*)*,* B-cell lymphoma 2-associated X (*BAX*), and B-cell lymphoma 2 (*BCL2*) in non-treated (negative control) and 10 and 100 ng/ml rhBMP-7-treated NPC pellets. Data are expressed as relative expression ± SD, except for the *BAX/BCL2* ratio. **Indicates significant difference at a 99% confidence interval (CI); *indicates significant difference at a 98% CI; δ indicates a significant difference at a 97% CI; filled circle indicates a significant difference at a 96% CI.

Expression of extracellular matrix genes *ACAN* and *COL2A1* was significantly upregulated at day 7 in the 100 ng/ml rhBMP-7-treated NPC pellets compared with the negative control and the 10 ng/ml rhBMP-7-treated NPC pellets (Figure 3A). *COL2A1* expression was significantly upregulated in the 100 ng/ml rhBMP-7-treated NPC pellets compared with the negative control at day 28. Expression of *COL1A1* did not differ between conditions at any time point. Relative expression of the proliferative marker *CCND1* was significantly upregulated in all conditions at day 28 compared with day 7, while it was also significantly higher in the 100 ng/ml rhBMP-7-treated NPC pellets at day 7 compared with the negative control. Furthermore, *CCND1* expression levels were significantly higher in the 10 ng/ml rhBMP-7-treated NPC pellets at day 28 compared with the negative control.

Gene expression of the catabolic gene *ADAMTS5* was significantly lower in the 100 ng/ml rhBMP-7-treated NPC pellets compared with the 10 ng/ml rhBMP-7-treated NPC pellets at day 28. In the NPC pellets treated with 10 and 100 ng/ml rhBMP-7, gene expression of the catabolic gene *MMP13* was significantly lower at day 28 compared with day 7. The relative gene expression of the anti-catabolic gene *TIMP1* was significantly higher in the negative control and 10 ng/ml rhBMP-7-treated NPC pellets at day 7 compared with day 28. The *BAX/BCL2* ratio in the 100 ng/ml rhBMP-7-treated NPC pellets was significantly higher at day 28 compared with day 7. Relative expression of the apoptotic marker *CASP3* was significantly downregulated at day 28 in the 10 and 100 ng/ml rhBMP-7-treated NPC pellets. Altogether, these results show a stimulatory effect of 100 ng/ml rhBMP-7 on matrix anabolism, which seemed most profound at day 7, and an anti-apoptotic effect, which seemed most profound at day 28.

### Intradiscal application of rhBMP-7 in laboratory beagle dogs

#### No regenerative changes of rhBMP-7 at the intervertebral disc level on macroscopy and T2-weighted MRI

All dogs recovered uneventfully from surgery and were ambulant the next day. During the follow-up period, all dogs were treated with analgesics and antibiotics in accordance with the protocol described under ‘Surgical procedure’, and none of the dogs required additional medication. Before surgery, a total of 63 IVDs were graded on T2W MR images (t_0_). All but one IVDs (62 out of 63) were assigned a grade II according to the Pfirrmann system, whereas 1 IVD was assigned a grade III. A total of 42 grade II IVDs were injected. In 61 IVDs the Pfirrmann scores remained unchanged over time. The IVD that was assigned a grade III at t_0_ was assigned a grade II at all subsequent time points, and one IVD treated with the sucrose buffer that was scored a grade II at t_0_, was re-graded a Pfirrmann score III. Postmortem, 62 out of 63 IVDs were assigned a Thompson score grade II, consistent with early IVD degeneration, whereas 1 IVD, treated with 250 μg rhBMP-7, was assigned a grade IV.

#### Extradiscal new bone formation after intradiscal injection of 25 μg and 250 μg rhBMP-7

**Figure 4:**
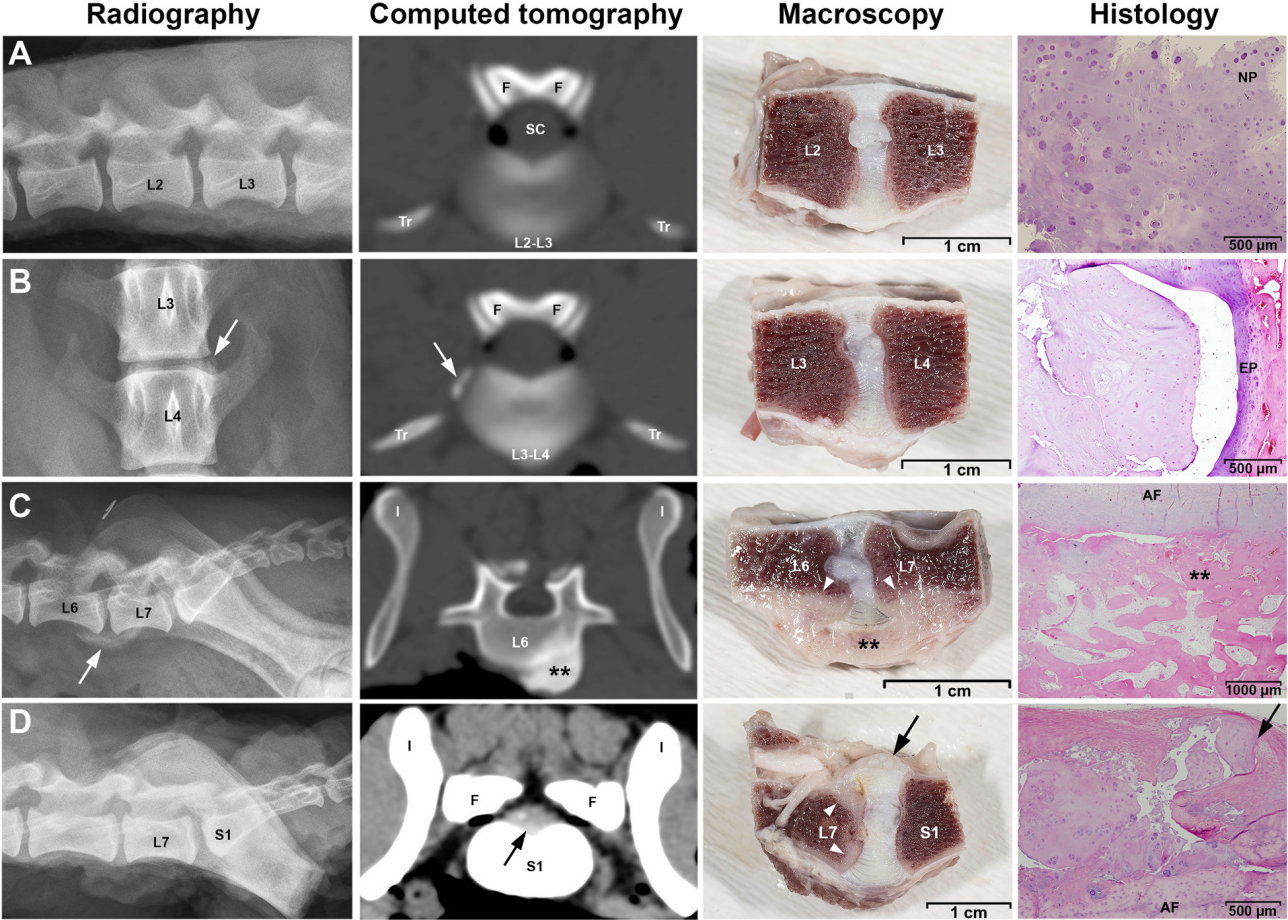
Extradiscal bone formation after intradiscal injection of rhBMP-7. Radiography, computed tomography (CT), macroscopy, and histology of intervertebral discs (IVDs) injected with 2.5 μg **(A)**, 25 μg **(B)**, and 250 μg **(C, D)** rhBMP-7. **A**. Unremarkable findings after injecting 2.5 μg rhBMP-7 into the IVD. The histological image shows a typical example of an early degenerated NP, consisting of clusters of chondrocyte-like cells. **B**. On the left lateral site of the vertebral column extradiscal new bone formation is shown on the radiograph and CT image (white arrow) next to the IVD injected with 25 μg rhBMP-7. Histology was consistent with early degenerative changes in the IVD as shown in A. **C**. Extradiscal new bone formation ventrally of the vertebral column and sclerosis of the ventral vertebral cortex is shown on radiography (white arrow), CT, macroscopy, and histology (**) after injecting 250 μg rhBMP-7 into the IVD. **D.** Rupturing of the dorsal AF and protrusion of NP material into the spinal canal (black arrow) on CT, macroscopy and histology image of an IVD injected with 250 μg rhBMP-7. Irregularity of the endplates and focal sclerosis in the subchondral bone (white arrowheads) was seen on macroscopy and confirmed on histology (not shown in this image). AF = annulus fibrosus, EP = endplate, F = facet joint, I = ilium, L = lumbar vertebra, NP = nucleus pulposus, S = sacral vertebra, SC = spinal cord, Tr = transverse process.

In one IVD, extensive new bone formation was noted on the ventral aspect on MRI at 6, 12, and 24 weeks post-surgery (t_6,_ t_12,_ and t_24_). Ventral bone formation was noted on the post-mortem macroscopy in this particular IVD and in one other. The IVD that was scored a Thompson grade IV showed rupturing of the dorsal AF, with NP material extending into the spinal canal, irregularity of the endplates, and focal sclerosis in the subchondral bone (Figure 4). CT images of this dog showed symmetric extensive bulging of the L7-S1 IVD (lumbosacral junction) that had been injected. In 4 out of 63 IVDs, postmortem radiographs and CT images revealed marginated extradiscal new bone formation ventrally, laterally (left side), and ventrolaterally (right side), in combination with mild sclerosis of the underlying bone (Figure 4). These findings were associated with treatment with 250 μg rhBMP-7 in 3 out of 42 IVDs, and treatment with 25 μg rhBMP-7 in 1 out of 42 IVDs. Mineralization was shown on CT images of 2 out of 63 IVDs; one of these IVDs had been injected with 2.5 μg rhBMP-7, whereas the other had not been injected. DHI, T2 values, and T1ρ values did not show significant differences between treatments over time.

#### Anti-apoptotic effect of 250 μg rhBMP-7

**Figure 5:**
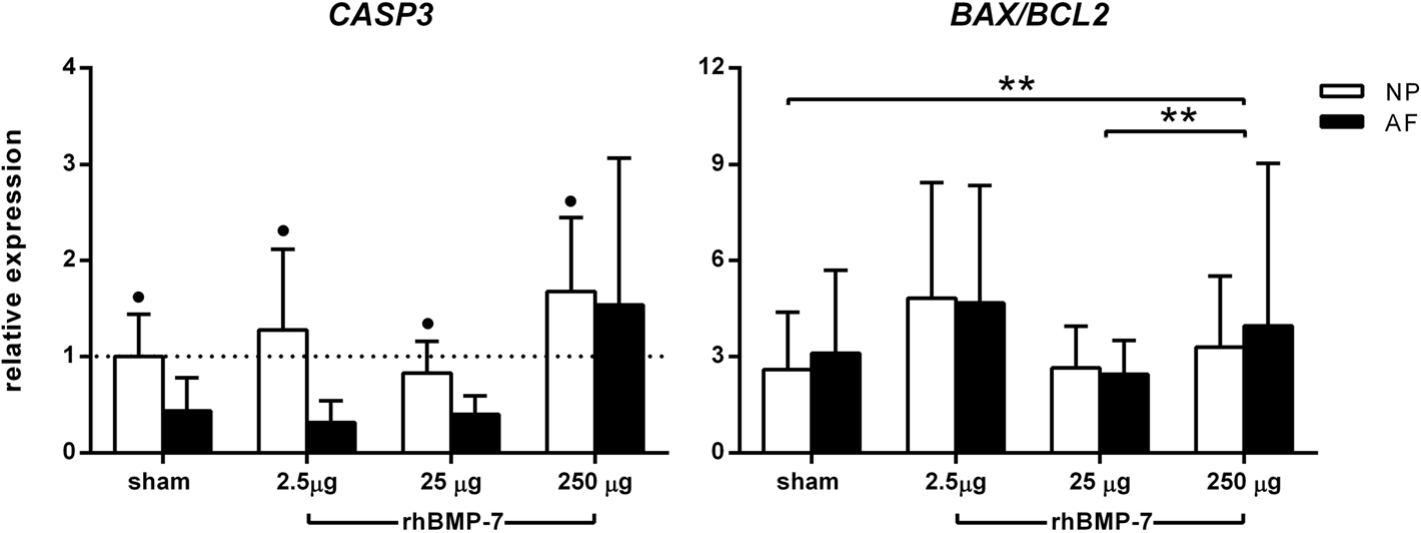
Relative gene expression of genes associated with apoptosis. Relative gene expression of caspase 3 (*CASP3*), B-cell lymphoma 2-associated X*/*B-cell lymphoma 2 (*BAX/BCL2*) ratio in the nucleus pulposus (NP) (open bars) and the annulus fibrosus (AF) (filled bars) injected with a sucrose buffer (sham), 2.5 μg, 25 μg, or 250 μg rhBMP-7, were indicative of an anti-apoptotic effect of 250 μg rhBMP-7. The sham-treated NP was used as a reference and was set at 1 (dashed line). Data are expressed as n-fold change ± SD. ** Indicates a significant difference at a 99% confidence interval (CI); filled circle indicates a significant difference at a 96% CI.

All IVDs were evaluated histopathologically according to the grading scheme according to Bergknut *et al*. [21]. The median histological grade was 13 (range: 8 to 20) and there were no significant differences between the treatments. In the IVD treated with 250 μg rhBMP-7 that was scored a Thompson grade IV, NP material and fibroblasts were present in the outer AF. Furthermore, bone formation was confirmed in 2 IVDs treated with 250 μg rhBMP-7. The *BAX/BCL2* ratio in the IVDs (NP and AF) treated with 250 μg rhBMP-7 was significantly upregulated compared with the IVDs (NP and AF) treated with the sham and 25 μg rhBMP-7, suggestive of an anti-apoptotic effect of 250 μg rhBMP-7. Relative gene expression of *CASP3* in the NP was significantly higher than in the AF in all treatments, indicative of a higher apoptotic rate in the NP (Figure 5).

#### No anabolic effects of rhBMP-7 on extracellular matrix

**Figure 6:**
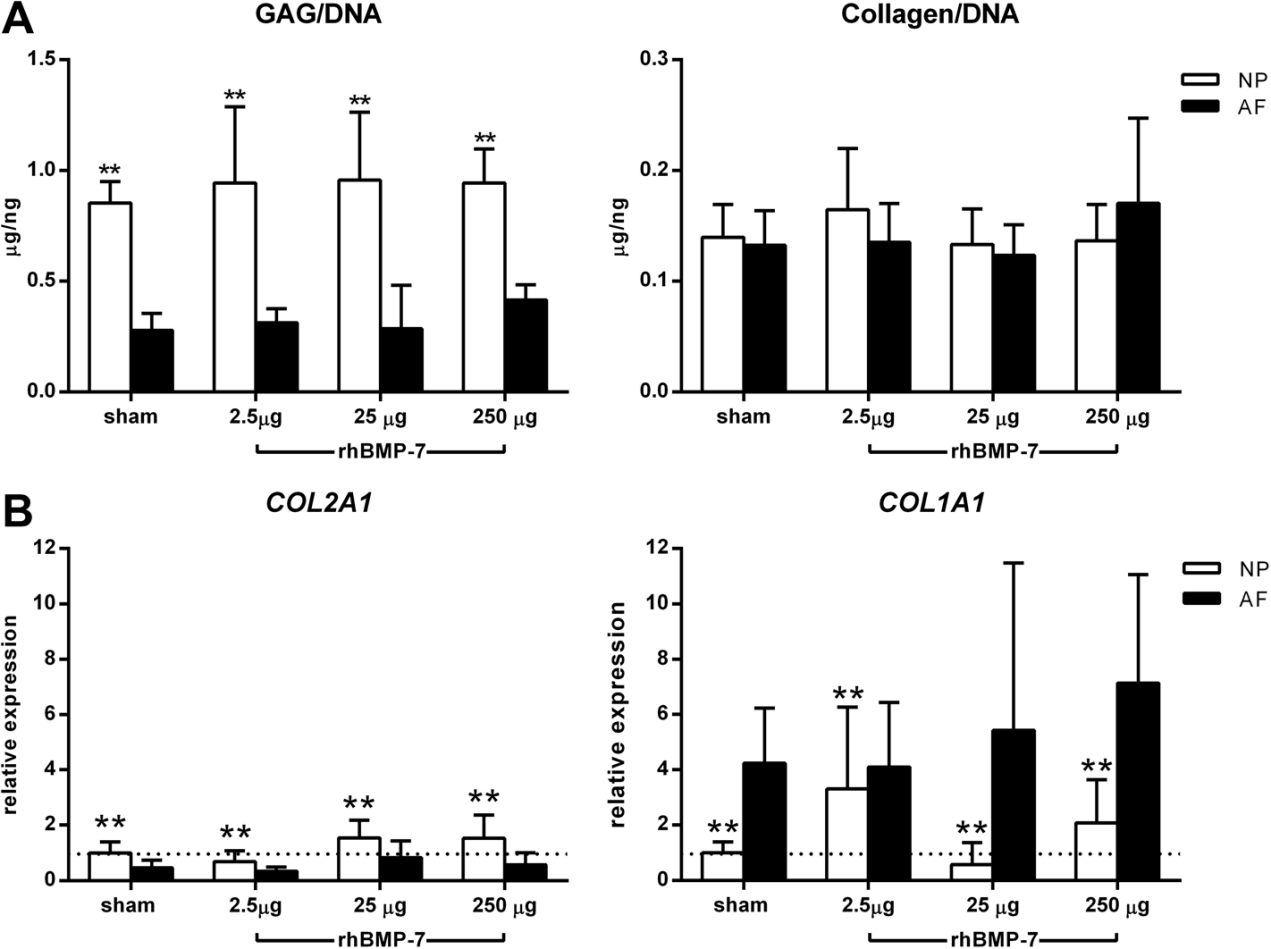
Relative gene expression of matrix-related target genes and DNA, glycosaminoglycan (GAG), and collagen content in intervertebral discs (IVDs) injected with rhBMP-7. **A**. GAG/DNA and collagen/DNA did not significantly differ between the treatments. GAG/DNA in the nucleus pulposus (NP) (open bars) was significantly higher than in the annulus fibrosus (AF) (filled bars). **B**. Relative gene expression of collagen type 2 alpha 1 (*COL2A1*) and collagen type 1 alpha 1 (*COL1A1*) did not significantly differ between treatments. Gene expression levels of *COL2A1* were significantly higher in the NP than in the AF, whereas the expression levels of *COL1A1* were significantly lower in the NP than in the AF. GAG/DNA and collagen/DNA are expressed as mean ± SD, and *COL2A1* and *COL1A1* as relative expression ± SD. **Indicates a significant difference at a 99% confidence interval (CI).

No significant differences in GAG corrected for DNA or collagen corrected for DNA were found between the treatments in the NP or the AF (Figure 6A). Regardless of the treatment, GAG/DNA in the NP was significantly higher than in the AF, consistent with the known physiological differences in matrix composition at protein level between the NP and the AF. These physiological differences were also reflected by relative gene expression, as gene expression levels of *COL2A1* were significantly higher in the NP than in the AF, and the expression levels of *COL1A1* were significantly lower in the NP than in the AF (Figure 6B). Relative gene expression of anabolic (*ACAN*, *COL2A1*, *COL1A1*), catabolic (*MMP13*) and anti-catabolic (*TIMP1*) genes did not significantly differ between treatments. Gene expression of *ADAMTS5* and BMP receptors *BMPR1A* and *BMPR1B* were below the detection level in both the NP and the AF, independent of treatment. BMP-7 receptor *BMPR2*, and the downstream target of the BMP-7 signaling pathway gene *ID1*, did not significantly differ between the treatments. Relative gene expression of BMP signaling inhibitor *NOG* varied widely regardless of the group, and was significantly higher in IVDs treated with 25 μg compared with 2.5 μg and sham-treated IVDs; relative gene expression of *NOG* is reported in Additional file 4.

## Discussion

In this study we first showed that 100 ng/ml rhBMP-7 was biologically active in vitro in canine NPCs isolated from mildly, spontaneously degenerated IVDs. Treatment with 100 ng/ml rhBMP-7 stimulated matrix production by canine NPC pellets isolated from degenerated discs, reflected by a significantly higher expression of *ACAN* and *COL2A1* seen at day 7, and a significant increase in GAG release and deposition mainly seen at day 28 of culture. Treatment with 10 ng/ml rhBMP-7 showed a significant increase in GAG release compared with the negative control, but did not result in increased GAG deposition. This is most likely due to a suboptimal balance in GAG and collagen synthesis and breakdown, resulting in an inability to deposit GAGs in the newly formed collagen network. During pellet culture, in the absence of rhBMP-7, DNA content decreased significantly over time. NPC pellets treated with rhBMP-7 showed preservation of the DNA content at initial levels, with a significant upregulation of the biomolecular proliferative marker *CCND1* limited to day 7, and a significant downregulation of the pro-apoptotic marker *CASP3* and an increase of the *BAX/BCL2* ratio at day 28. Our findings are consistent with the pro-anabolic and anti-apoptotic properties of rhBMP-7 that have been shown *in vitro* in NP cells of different species, such as bovine, rabbit, and human [8,12,13,34,35].

Next, we aimed at determining the safe and optimal regenerative dose of rhBMP-7 for intradiscal application in a spontaneous canine disc degeneration model. However, intradiscal injection of a wide dose range of rhBMP-7 (2.5 to 250 μg) did not result in regeneration of the canine IVD. In contrast to what has been described in rabbit models, extracellular matrix production in the NP and the AF did not differ between treatments. It should be noted that our data analysis is limited by the absence of wet weight data for the samples, and that the necessary correction for DNA content might have leveled out the effects between treatments. However, DHI, T2, and T1ρ maps and histological grading did not differ between treatments, confirming the biochemical data and corroborating the absence of a regenerative effect after intradiscal application of a wide range of dosages of rhBMP-7. Contrasting findings between the current canine model based on spontaneous degeneration and the canine models or rabbit models of induced IVD degeneration, are most likely related to differences in disc size and cell types that populate the NP. Cell type variation related to differences in genetic background may play a role, with a concomitant difference in degenerative, as well as regenerative pathways. Notochordal cells (NCs) are thought to play a key role in regeneration [36]. In rabbits, NCs are retained in the NP at least until 12 months of age, while in humans NCs are lost before 10 years of age, and in chondrodystrophic dogs, such as beagles, before one year of age [37]. Although induction of rabbit IVD degeneration by trauma is accompanied by an inflammatory response and ultimately results in decreased amounts of extracellular matrix, a clear loss in disc height, and replacement of NCs by chondrocyte-like and fibroblast-like cells, the persistence of NCs cannot be ruled out.

Nevertheless, early degenerated canine NP cells in culture respond in a similar way to rhBMP-7 as rabbit NP cells, also suggestive of differences in rhBMP-7 activity *in vitro* and *in vivo* in the canine model. The anabolic and anti-apoptotic effect of rhBMP-7 on the tissue cells is mediated via specific BMP receptors that activate the intracellular signaling protein SMAD1/5/8. The latter then forms a complex with SMAD4, and the complex translocates into the nucleus and regulates the transcription of target genes. BMP-7, in addition, upregulates natural BMP antagonists such as *NOG* that block the binding sites of the BMP receptors, thereby bringing rhBMP-7 in an inactive state [38]*.* Relative gene expression of *NOG* was significantly upregulated in IVDs treated with 25 μg versus 2.5 μg and sham-treated IVDs, indicating that BMP antagonists may indeed play a role in the regulation of *in vivo* BMP-7 signaling. Given that the BMP signaling pathway is complicated, we can only speculate on the possible confounding effects limiting the biological effect of rhBMP-7 *in vivo* after six months of follow-up, including availability of BMP receptors, activity of *NOG*, and the presence and ability of proteases in the degenerated IVD to degrade rhBMP-7. Another explanation for the differences *in vitro* and *in vivo* was the addition of rhBMP-7 biweekly *in vitro*, while a single dose was applied intradiscally *in vivo*. Protein activity *in vivo* is likely to be lost quickly due to a short biological half-life and diffusion out of the tissue [39]. The bioavailability of rhBMP-7 *in vivo* could be increased by using controlled-release systems, such as hydrogels or microspheres which allow a sustained release of rhBMP-7 over a prolonged period of time, or by vector-mediated introduction of BMP-7 encoding genes.

Strikingly, mild to severe extradiscal new bone formation was seen after intradiscal administration of 25 μg and 250 μg rhBMP-7 in our canine model. Induction of bone formation requires three essential components: an osteo-inductive signal, a substratum, and interactive host cells. The surgical procedure, associated with tissue injury, might have provided the chemotactic stimulus for the recruitment of required mesenchymal precursor cells. The application of rhBMP-7 might have provided the osteo-inductive stimulus for chondro-osteogenic differentiation, resulting in ectopic bone formation. Although suboptimal delivery of BMP-7 by our injection technique might have played a role, the rhBMP-7 might also have diffused out of the NP. This phenomenon was also previously described in a rabbit model, in which osteophyte formation was induced by intradiscal injection of labelled mesenchymal stem cells [40]. Diffusion of rhBMP-7 out of the IVD may have been enhanced by biomechanical forces and/or disorganization of the lamellar structure of the AF that are part of the early IVD degeneration process [2]. Various dosages of rhBMP-7, ranging from 0.005 μg to 2 mg, with or without a carrier, have been reported to induce endochondral bone formation in extraskeletal sites (muscle, subcutis, tendon, thyroid cartilage, and subdural space) and in several species (for example, baboons, rats, and dogs) [6,41-44]. In all these studies, the microenvironment appeared to be an essential component in tissue regeneration. Indeed, rhBMP-7 is approved for the treatment of non-unions of long bones and the pelvis, and for posterior lumbar fusion in humans [45]. rhBMP-7 is frequently used in humans, however, adverse effects, such as ectopic bone formation, are only occasionally reported [45-47]. Based on statements of researchers in reviews from 2008 and 2011, a multicenter clinical trial in the United States was started, in which BMP-7 was intradiscally injected into human patients with degenerative disc disease [48,49]. However, results have not yet been published.

## Conclusions

An anabolic effect of rhBMP-7 on extracellular matrix production of canine NPCs isolated from early degenerated IVDs was shown *in vitro*. Despite intradiscal administration of a wide dose range of rhBMP-7 (2.5 to 250 μg) in spontaneous early degenerated canine IVDs, we did not observe regenerative effects at the IVD level. In fact, injection of 250 μg rhBMP-7 and, to a lesser extent 25 μg rhBMP-7, resulted in extensive extradiscal bone formation. Altogether, this study indicates that additional issues need to be addressed before intradiscally applied rhBMP-7 can be translated into a clinical application to treat low back pain in human and canine patients.

## Additional files

Additional file 1:
**Primers used for quantitative PCR.**
Additional file 2:
**Biological activity of recombinant human BMP-7.** Induction of ALP activity by rhBMP-7 from two different manufacturers *in vitro* and by the rhBMP-7 dialysate *in vitro* [50,51]. Additional file 3:
**Significant differences and the corresponding confidence intervals.** Tables S1 and S2 represent significant differences and confidence intervals of statistical analyses performed on *in vitro* and *in vivo* data, respectively. Additional file 4:
**Relative gene expression of noggin in IVDs injected with rhBMP-7.**

## Abbreviations

*ACAN*: aggrecan
*ADAMTS5*: a disintegrin and metalloproteinase with thrombospondin motifs 5
AF: annulus fibrosus
AIC: Akaike Information Criterion
ALP: alkaline phosphatase
ATDC5: AT805 teratocarcinoma derived chondrogenic cells
*BAX*: B-cell lymphoma 2-associated X
*BCL-2*: B-cell lymphoma 2
bFGF: basic fibroblast growth factor
BLAST: Basic Local Alignment Search Tool
BMP: bone morphogenetic protein
*BMPR1A*: BMP receptor 1A
*BMPR1B*: BMP receptor 1B
*BMPR2*: BMP receptor 2
*CASP3*: caspase 3
*CCND1*: cyclin-D1
*COL1A1*: collagen type 1 alpha 1
*COL2A1*: collagen type 2 alpha 1
Ct: computed tomography
Ct: cycle threshold
DEC: Animal Experiments Committee (Dutch: Dierexperimentencommissie)
DHI: disc height index
DMMB: dimethylmethylene blue
DMSO: dimethyl sulfoxide
ECM: extracellular matrix
FBS: fetal bovine serum
FOV: field of view
GAG: glycosaminoglycan
*GAPDH*: glyceraldehyde 3-phosphate dehydrogenase
hg-DMEM: high-glucose Dulbecco’s modified Eagle’s medium
*HPRT*: hypoxanthine-guanine phosphoribosyltransferase
*ID1*: inhibitor of DNA binding
ITS: insulin-transferrin-selenium
IVD: intervertebral disc
*MMP13*: matrix metalloproteinase 13
MRI: magnetic resonance imaging
NC: notochordal cells
*NOG*: noggin
NP: nucleus pulposus
NPC: nucleus pulposus cells
p/s: penicillin/streptomycin
P2: passage 2
qPCR: real-time quantitative polymerase chain reaction
rhBMP-7: recombinant human bone morphogenetic protein 7
ROI: region of interest
*RPS19*: ribosomal protein S19
S: signal intensity
*SDHA*: succinate dehydrogenase complex, subunit A, flavoprotein variant
T1-TFE: T1-turbo field echo
TE: echo time
TGF-β: transforming growth factor-β
*TIMP1*: tissue inhibitor of metalloproteinase 1
TR: repetition time
TSE: turbo-spin echo
TSL: spin-lock time
