# Supplementary material for: Intradiscal application of rhBMP-7 does not induce regeneration in a canine model of spontaneous intervertebral disc degeneration
Source: Arthritis Res Ther. 2015 May 27;17(1):137. doi: 10.1186/s13075-015-0625-2 (PMC4443547; doi:10.1186/s13075-015-0625-2)
Supplement: Additional file 1: — Primers used for quantitative PCR. [file 13075_2015_625_MOESM1_ESM.doc]

**Additional file 1. *Primers used for quantitative PCR***

| **Protein** | **Forward sequence 5’ -> 3’** | **EXON** | **Reverse sequence 5’ -> 3’** | **EXON** | **Amplicon size** | **Annealing temp (°C)** | **Accession no.** |
| --- | --- | --- | --- | --- | --- | --- | --- |
| **Reference genes** |  |  |  |  |  |  |  |
| GAPDH | TGTCCCCACCCCCAATGTATC | 7 | CTCCGATGCCTGCTTCACTACCTT | 8 | 100 | 58 | NM_001003142 |
| RPS19 | CCTTCCTCAAAAAGTCTGGG | 2/3 | GTTCTCATCGTAGGGAGCAAG | 3 | 95 | 61 | XM_005616513 |
| SDHA | GCCTTGGATCTCTTGATGGA | 6 | TTCTTGGCTCTTATGCGATG | 6 | 92 | 61 | DQ_402985 |
| HPRT | AGCTTGCTGGTGAAAAGGAC | 5/6 | TTATAGTCAAGGGCATATCC | 7 | 104 | 58 | NM_001003357 |
| **Target genes** |  |  |  |  |  |  |  |
| ACAN | GGACACTCCTTGCAATTTGAG | 13/14 | GTCATTCCACTCTCCCTTCTC | 14 | 111 | 62 | XM_005618252 |
| COL2A1 | GCAGCAAGAGCAAGGAC | 52 | TTCTGAGAGCCCTCGGT | 53 | 151 | 62 | XM_005636674 |
| COL1A1 | GTGTGTACAGAACGGCCTCA | 2 | TCGCAAATCACGTCATCG | 2 | 109 | 61 | NM_001003090 |
| ADAMTS5 | CTACTGCACAGGGAAGAG | 5 | GAACCCATTCCACAAATGTC | 6 | 149 | 61 | XM_846025.3 |
| MMP13 | CTGAGGAAGACTTCCAGCTT | 1 | TTGGACCACTTGAGAGTTCG | 2 | 250 | 65 | XM_536598 |
| TIMP1 | GGCGTTATGAGATCAAGATGAC | 2 | ACCTGTGCAAGTATCCGC | 3 | 120 | 66 | NM_001003182 |
| CCND1 | GCCTCGAAGATGAAGGAGAC | 1 | CAGTTTGTTCACCAGGAGCA | 1 | 151 | 60 | NM_001005757 |
| BMPR1A | TTTGGGAAATGGCTCGTC | ?? | CGTATGATGGATCGTTGGG | ?? | ?? | 60 | NM_004329.2 |
| BMPR1B | CCCTATCATGACCTAGTGCC | ?? | TGCCTCAGACACTCATCAC | ?? | ?? | 63 | NM_001203.2 |
| BMPR2 | AGAGACCCAAGTTCCCAG | ?? | CATCATAAGTTCAGCCATCCT | ?? | ?? | 60 | ?? |
| ID1 | CTCTACGACATGAACGGCTGT | ?? | TGCTCACCTTGCGGTTCTG | ?? | ?? | 62 | ?? |
| NOG | TGCCGAGCGAGATCAAAGGG | 2 | AGCCACATCTGTAACTTCCTCCG | 2 | 99 | 63 | ?? |
| BAX | CCTTTTGCTTCAGGGTTTCA | 2/3 | CTCAGCTTCTTGGTGGATGC | 3 | 108 | 59 | NM_001003011 |
| BCL2 | TGGAGAGVGTCAACCGGGAGATGT | 3 | AGGTGTGCAGATGCCGGTTCAGGT | 3 | 87 | 62 | NM_001002949 |
| CASP3 | ATCACTGAAGATGGATGGGTTGGGTT | 8 | TGAAAGGAGCATGTTCTGAAGTAGCACT | 8 | 139 | 58 | NM_001003042 |

Primers used for qPCR analysis of reference genes *glyceraldehyde 3-phosphate dehydrogenase* (*GAPDH*), *ribosomal protein S19* (*RPS19*), s*uccinate dehydrogenase complex, subunit A, flavoprotein variant* (*SDHA)*, and *hypoxanthine-guanine phosphoribosyltransferase* *(HPRT),* and target genes *aggrecan (ACAN), collagen type II (COL2A1), collagen type I (COL1A1)*, *a disintegrin and metalloproteinase with thrombospondin motifs (ADAMTS5), matrix metalloproteinase 13 (MMP13), tissue inhibitor of metalloproteinase 1 (TIMP1*), *cyclin-D1 (CCND1)*, *bone morphogenetic protein receptor type IA (BMPR1A), bone morphogenetic protein receptor type IB (BMPR1B), bone morphogenetic protein receptor type II (BMPR2),* *DNA-binding protein inhibitor 1 (ID1),* *noggin (NOG)*, *B-cell lymphoma 2-associated X (BAX), B-cell lymphoma 2 (BCL2) and caspase 3 (CASP3)*.
