# Supplementary material for: Intradiscal application of rhBMP-7 does not induce regeneration in a canine model of spontaneous intervertebral disc degeneration
Source: Arthritis Res Ther. 2015 May 27;17(1):137. doi: 10.1186/s13075-015-0625-2 (PMC4443547; doi:10.1186/s13075-015-0625-2)
Supplement: Additional file 2: — Biological activity of recombinant human BMP-7. Induction of ALP activity by rhBMP-7 from two different manufacturers in vitro and by the rhBMP-7 dialysate in vitro [50,51]. [file 13075_2015_625_MOESM2_ESM.doc]

**Additional file 2. *Biological activity of rhBMP-7***

***Induction of ALP activity by rhBMP-7 from two different manufacturers in vitro***


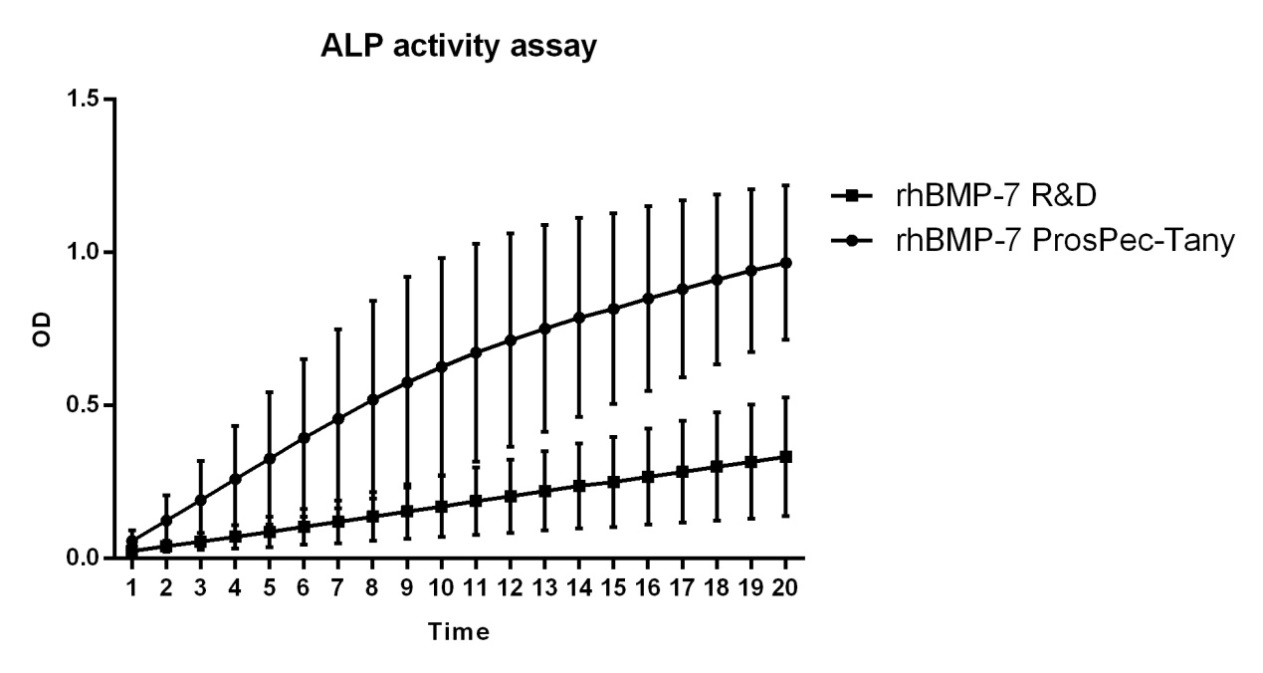
rhBMP-7 of manufacturers R&D and ProsPec-Tany were evaluated for their activity to induce ALP activity in mice ATDC5 cells. ATDC5 cells were cultured as described by Caron et al. and previously reported by us [50, 51]. Cells were plated on 24-well plates (Greiner Cellstar, Alphen a/d Rijn, The Netherlands) at a density of 4*10^4^ cells/plate. After 24 hours, standard differentiation culture medium with 100 ng/ml BMP-7 was added, and cells were cultured for another 72 hours and total ALP activity was measured kinetically in the cell lysate in a kinetic microplate reader at 405 nm (Bio-Rad Benchmark, Veenendaal, The Netherland). A volume of 50 μl of the cell lysate incubated was with 50 μl p-nitrophenyl phosphate for 20 minutes. rhBMP-7 of ProsPec-Tany induces a higher ALP activity compared with rhBMP-7 of R&D at the same concentration (Figure 1).

**Figure 1.** rhBMP-7 of ProsPec-Tany induces a higher ALP activity compared with rhBMP-7 of R&D at the same concentration in ATDC5 cells undergoing differentiation.

***Induction of ALP activity by rhBMP-7 dialysate in vitro***After dialysis of the rhBMP-7 (ProsPec-Tany) biologic activity of the dialysate was determined by induction of ALP activity in mice ATDC5 cells (ProsPec-Tany) and compared to pre-dialyzed BMP-7 at the same concentration. As depicted in figure 2, the dialysate of ProsPec-Tany retained its biological activity.

***
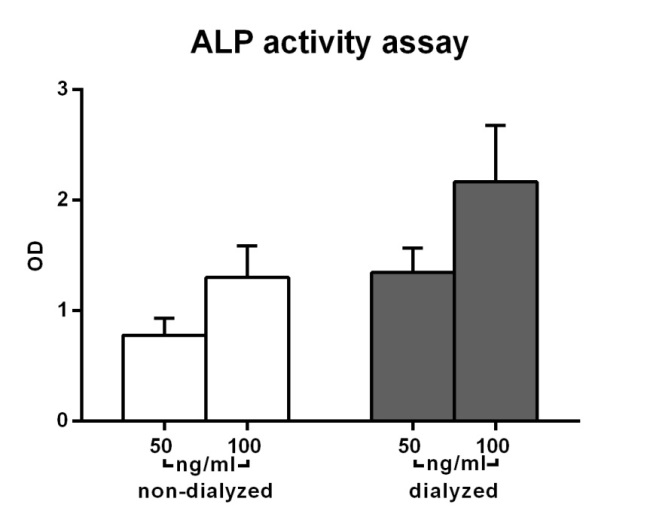
***

**Figure 2.** Dialyzed rhBMP-7 retained its biological activity at similar levels as the non-dialyzed product.
