# Supplementary material for: Intradiscal application of rhBMP-7 does not induce regeneration in a canine model of spontaneous intervertebral disc degeneration
Source: Arthritis Res Ther. 2015 May 27;17(1):137. doi: 10.1186/s13075-015-0625-2 (PMC4443547; doi:10.1186/s13075-015-0625-2)
Supplement: Additional file 3: — Significant differences and the corresponding confidence intervals. Tables S1 and S2 represent significant differences and confidence intervals of statistical analyses performed on in vitro and in vivo data, respectively. [file 13075_2015_625_MOESM3_ESM.doc]

**Additional file 3. *Significant differences and the corresponding confidence intervals***

Table 1 and 2 represent significant differences and confidence intervals of statistical analyses performed on *in vitro* and *in vivo* data, respectively. Figures correspond to figures shown in the main article.

| ***In vitro* experiment** | ***Figure 1. DNA, GAG and GAG/DNA content in pellets containing nucleus pulposus cells.*** | | | | | |
| --- | --- | --- | --- | --- | --- | --- |
| ***Cumulative GAG release*** |  | | | | | |
| **Condition** | **vs** | **Condition** | **Difference in location*** | **Confidence interval (CI)** | **CI (%)** | ***p-*value** |
| - control |  | 10 ng/ml rhBMP-7 | -25.40 | - 44.51 – -3.93 | 99 | 0.008 |
| - control |  | 100 ng/ml rhBMP-7 | -125.26 | -165.73 – -88.63 | 99 | 0.008 |
| 10 ng/ml rhBMP-7 |  | 100 ng/ml rhBMP-7 | -101.49 | -143.78 – -63.23 | 99 | 0.008 |
| ***DNA content*** |  | | | | | |
| **Condition** | **vs** | **Condition** | **Difference in location*** | **Confidence interval (CI)** | **CI (%)** | ***p-*value** |
| - control at day 7 |  | day 0 | 1.09 | 0.36 – 1.73 | 99 | 0.008 |
| 10 ng/ml rhBMP-7 at day 7 |  | day 0 | 1.07 | 0.23 – 1.76 | 99 | 0.008 |
| 100 ng/ml rhBMP-7 at day 7 |  | day 0 | 0.65 | 0.16 – 1.45 | 96 | 0.03 |
| - control at day 28 |  | day 0 | 1.41 | 0.91 – 2.00 | 99 | 0.009 |
| 10 ng/ml rhBMP-7 at day 28 |  | day 0 | 1.33 | 0.85 – 1.88 | 99 | 0.009 |
| 100 ng/ml rhBMP-7 at day 7 |  | day 0 | 0.93 | 0.21 – 1.52 | 99 | 0.008 |
| - control at day 7 |  | - control at day 28 | 0.27 | 0.09 – 0.73 | 99 | 0.009 |
| 10 ng/ml rhBMP-7 at day 7 |  | 10 ng/ml rhBMP-7 at day 28 | 0.54 | 0.04 – 0.74 | 98 | 0.01 |
| 10 ng/ml rhBMP-7 at day 28 |  | 100 ng/ml rhBMP-7 at day 28 | -0.40 | -0.76 – -0.24 | 99 | 0.009 |
| - control at day 28 |  | 100 ng/ml rhBMP-7 at day 28 | -0.30 | -0.88 – -0.30 | 99 | 0.009 |
| ***GAG content*** |  | | | | | |
| 10 ng/ml rhBMP-7 at day 28 |  | 100 ng/ml rhBMP-7 at day 28 | -6.41 | -13.74 – -1.27 | 99 | 0.008 |
| - control at day 28 |  | 100 ng/ml rhBMP-7 at day 28 | -6.42 | -13.74 – -1.80 | 99 | 0.007 |
| ***GAG/DNA*** |  | | | | | |
| 10 ng/ml rhBMP-7 at day 28 |  | 100 ng/ml rhBMP-7 at day 28 | -8.2 | -15.49 – -0.39 | 99 | 0.008 |
| - control at day 28 |  | 100 ng/ml rhBMP-7 at day 28 | -8.2 | -15.49 – -2.48 | 99 | 0.007 |
|  |  |  |  |  |  |  |

**Table 1A.** Significant differences and confidence intervals of statistical analyses performed on data of the *in vitro* experiment.

GAG = glycosaminoglycan, DNA = deoxyribonucleic acid **Difference in location estimates the median of the difference between condition x and condition y.*

|  | | | | | | | |
| --- | --- | --- | --- | --- | --- | --- | --- |
| ***In vitro experiment*** | | ***Figure 3. Relative gene expression of relevant target genes in nucleus pulposus cells cultured in pellets.*** | | | | | |
| ***qPCR analysis*** | |  | | | | | |
| **Condition** | | **vs** | **Condition** | **Difference in location*** | **Confidence interval (CI)** | **CI (%)** | ***p-*value** |
| *ACAN* | - control at day 7 |  | 100 ng/ml rhBMP-7 at day 7 | 2.67 | 0.74 – 3.90 | 99 | 0.008 |
|  | 10 ng/ml rhBMP-7 at day 7 |  | 100 ng/ml rhBMP-7 at day 7 | 2.17 | 0.45 – 4.01 | 99 | 0.008 |
| *COL2A1* | - control at day 7 |  | 100 ng/ml rhBMP-7 at day 7 | 4.15 | 0.94 – 5.51 | 99 | 0.009 |
|  | 10 ng/ml rhBMP-7 at day 7 |  | 100 ng/ml rhBMP-7 at day 7 | 3.70 | 1.42 – 5.54 | 99 | 0.009 |
|  | - control at day 28 |  | 100 ng/ml rhBMP-7 at day 28 | 3.92 | 0.84 – 7.92 | 96 | 0.03 |
| *CCND1* | - control at day 7 |  | 100 ng/ml rhBMP-7 at day 7 | 0.70 | 0.2 – 1.76 | 99 | 0.008 |
|  | - control at day 28 |  | 10 ng/ml rhBMP-7 at day 28 | 0.25 | 0.02 – 0.73 | 97 | 0.03 |
|  | - control at day 7 |  | - control at day 28 | 1.27 | 0.56 – 2.28 | 99 | 0.008 |
|  | 10 ng/ml rhBMP-7 at day 7 |  | 10 ng/ml rhBMP-7 at day 28 | 1.17 | 0.3 – 2.06 | 98 | 0.02 |
|  | 100 ng/ml rhBMP-7 at day 7 |  | 100 ng/ml rhBMP-7 at day 28 | 0.74 | 0.09 – 1.38 | 98 | 0.02 |
| *ADAMTS5* | 10 ng/ml rhBMP-7 at day 28 |  | 100 ng/ml rhBMP-7 at day 28 | -1.96 | -2.69 – -0.78 | 98 | 0.03 |
| *MMP13* | 10 ng/ml rhBMP-7 at day 7 |  | 10 ng/ml rhBMP-7 at day 28 | -3.62 | -6.72 – -0.27 | 98 | 0.02 |
|  | 100 ng/ml rhBMP-7 at day 7 |  | 100 ng/ml rhBMP-7 at day 28 | -2.81 | -3.86 – -1.16 | 98 | 0.02 |
| *TIMP1* | - control at day 7 |  | - control at day 28 | -2.57 | -3.34 – -0.91 | 99 | 0.009 |
|  | 10 ng/ml rhBMP-7 at day 7 |  | 10 ng/ml rhBMP-7 at day 28 | -1.84 | -3.16 – -0.00 | 96 | 0.04 |
| *CASP3* | - control at day 28 |  | 10 ng/ml rhBMP-7 at day 28 | -0.67 | -1.20 – -0.04 | 99 | 0.008 |
|  | - control at day 28 |  | 100 ng/ml rhBMP-7 at day 28 | -1.16 | -1.49 – -0.27 | 98 | 0.02 |
|  | - control at day 7 |  | - control at day 28 | 0.71 | 0.02 – 1.24 | 98 | 0.02 |
| *BAX/BCL2* | 100 ng/ml rhBMP-7 at day 7 |  | 100 ng/ml rhBMP-7 at day 28 | -0.14 | -0.26 – -0.01 | 96 | 0.04 |

**Table 1B.** Significant differences and confidence intervals of statistical analyses performed on data of the *in vitro* experiment.

**Difference in location estimates the median of the difference between condition x and condition y.
ACAN = aggrecan, COL2A1 = collagen type 2, CCND1 = cyclin-D1, ADAMTS5 = a disintegrin and metalloproteinase with thrombospondin motifs 5, MMP13 = matrix metalloproteinase 13, TIMP = tissue inhibitor of metalloproteinase 1, CASP = caspase 3, BAX = B-cell lymphoma 2-associated X, BCL2 = B-cell lymphoma 2*

| ***In vivo* experiment** | | ***Figure 5. Relative gene expression of genes associated with apoptosis*** | | | | | |
| --- | --- | --- | --- | --- | --- | --- | --- |
| ***qPCR analysis*** | |  | | | | | |
| **Condition** | | **vs** | **Condition** | **Hazard ratio (HR)** | **Confidence interval (CI)** | **CI (%)** | ***p-*value** |
| *CASP3* | NP |  | AF | 2.13 | 1.02 – 4.44 | 96 | 0.03 |
| *BAX/BCL2* | 250 μg rhBMP-7 |  | Sham | 7.05 | 1.07 – 12.52 | 98 | 0.02 |
|  | 25 μg rhBMP-7 |  | 250 μg rhBMP-7 | 0.26 | 0.08 – 0.89 | 99 | 0.005 |
|  | | ***Figure 6. Relative gene expression of matrix-related target genes and DNA, GAG, and collagen content in IVDs injected with rhBMP-7*** | | | | | |
| ***qPCR analysis*** | |  | | | | | |
| **Condition** | | **vs** | **Condition** | **Hazard ratio (HR)** | **Confidence interval (CI)** | **CI (%)** | ***p-*value** |
| *COL1A1* | NP |  | AF | 0.22 | 0.08 – 0.61 | 99 | 0.0001 |
| *COL2A1* | NP |  | AF | 2.46 | 1.07 – 5.65 | 99 | 0.006 |
|  | |  | | | | | |
| ***GAG/DNA*** | |  | | | | | |
| **Condition** | | **vs** | **Condition** | **Median** | **Confidence interval (CI)** | **CI (%)** | ***p-*value** |
| NP | |  | AF | 1.13 | 0.99 – 1.26 | 99 | <0.0001 |
|  | | ***Supplementary file 4. Relative gene expression of BMP antagonist noggin (NOG) in IVDs injected with rhBMP-7*** | | | | | |
| **Condition** | | **vs** | **Condition** | **Hazard ratio (HR)** | **Confidence interval (CI)** | **CI (%)** | ***p-*value** |
| *NOG* | 25 μg rhBMP-7 |  | 2.5 μg rhBMP-7 | 0.12 | 0.02 – 0.63 | 99 | 0.0009 |
|  | 25 μg rhBMP-7 |  | sham | 0.15 | 0.03 – 0.73 | 99 | 0.002 |

**Table 2.** Significant differences and confidence intervals of statistical analyses performed on data of the *in vivo* experiment.

*CASP = caspase 3, BAX = B-cell lymphoma 2-associated X, BCL2 = B-cell lymphoma 2, NOG = noggin*
