# Supplementary material for: Intradiscal application of rhBMP-7 does not induce regeneration in a canine model of spontaneous intervertebral disc degeneration
Source: Arthritis Res Ther. 2015 May 27;17(1):137. doi: 10.1186/s13075-015-0625-2 (PMC4443547; doi:10.1186/s13075-015-0625-2)
Supplement: Additional file 4: — Relative gene expression of noggin in IVDs injected with rhBMP-7. [file 13075_2015_625_MOESM4_ESM.doc]

**Additional file 4. *Relative gene expression of noggin in IVDs injected with rhBMP-7***

***
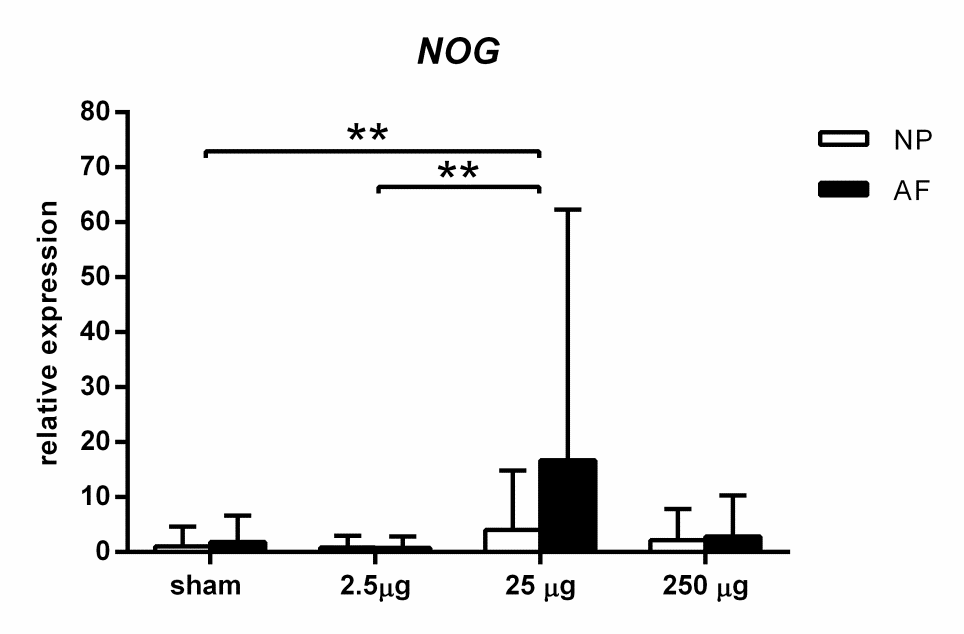
***

Relative gene expression of BMP antagonist noggin (NOG) in canine IVDs injected with rhBMP-7.

** *Indicates significant difference at a 99% confidence interval (CI);*
